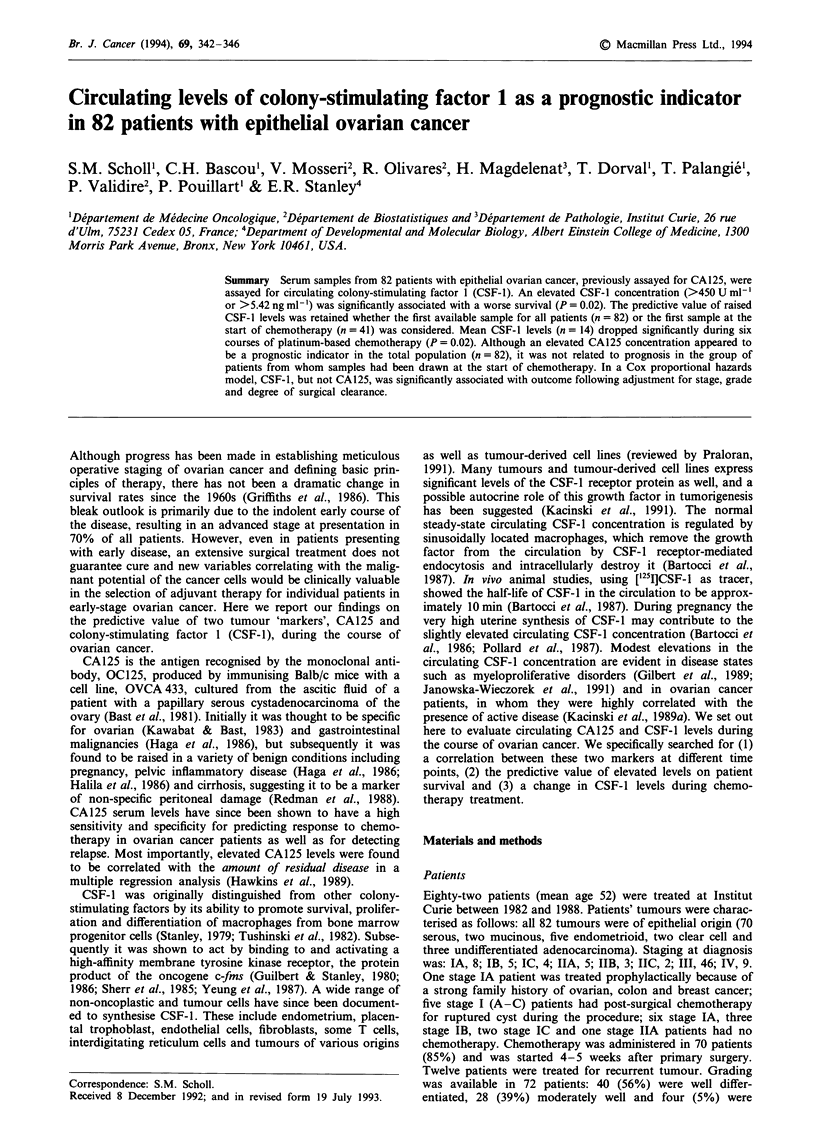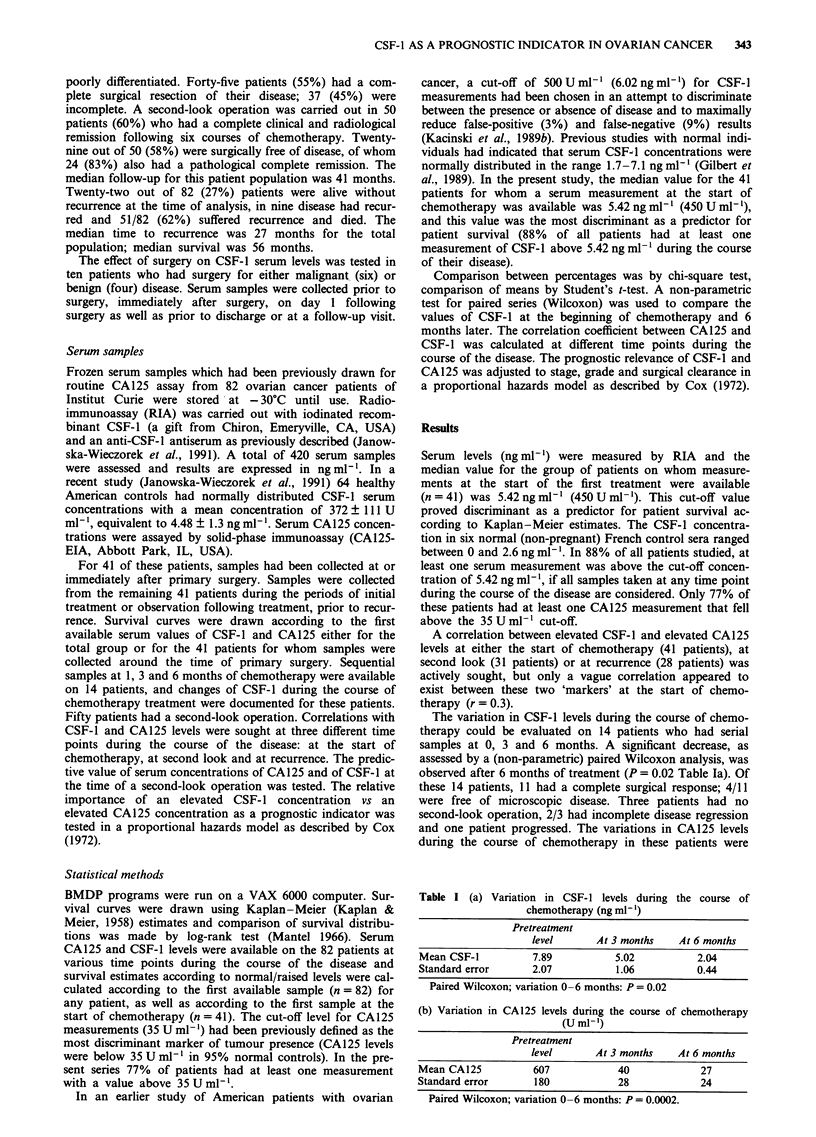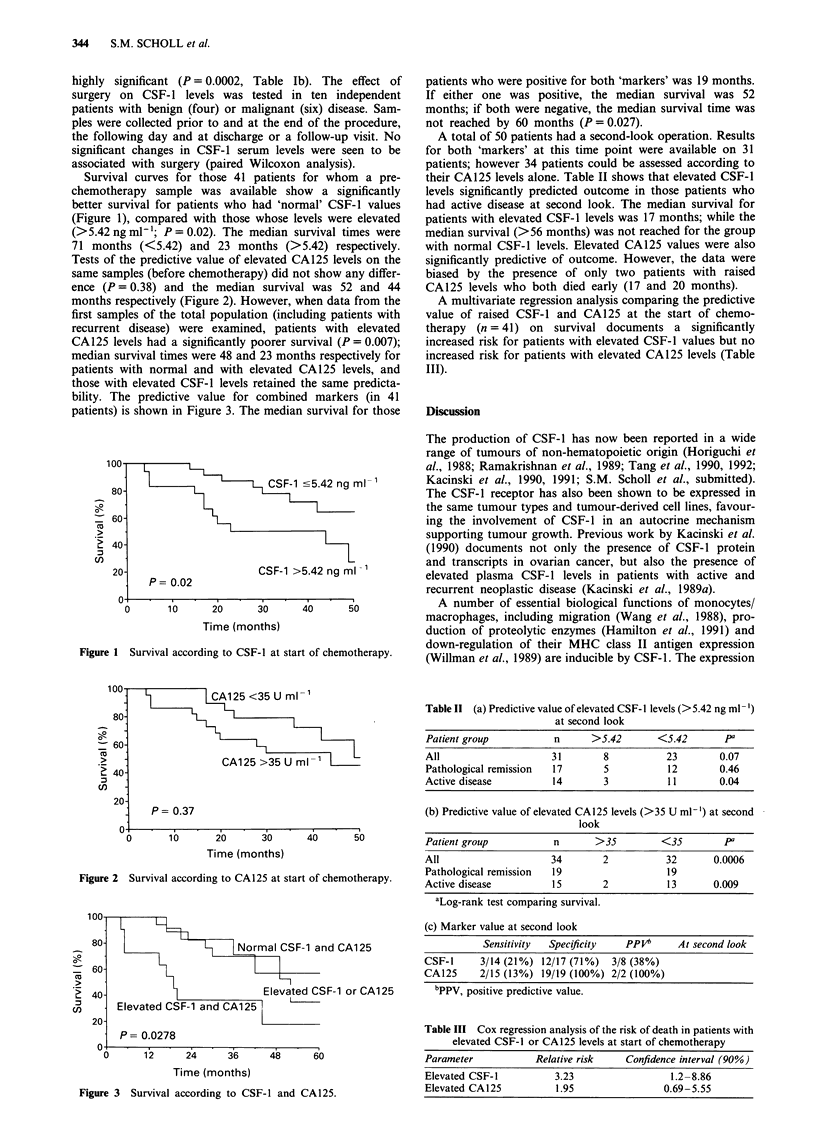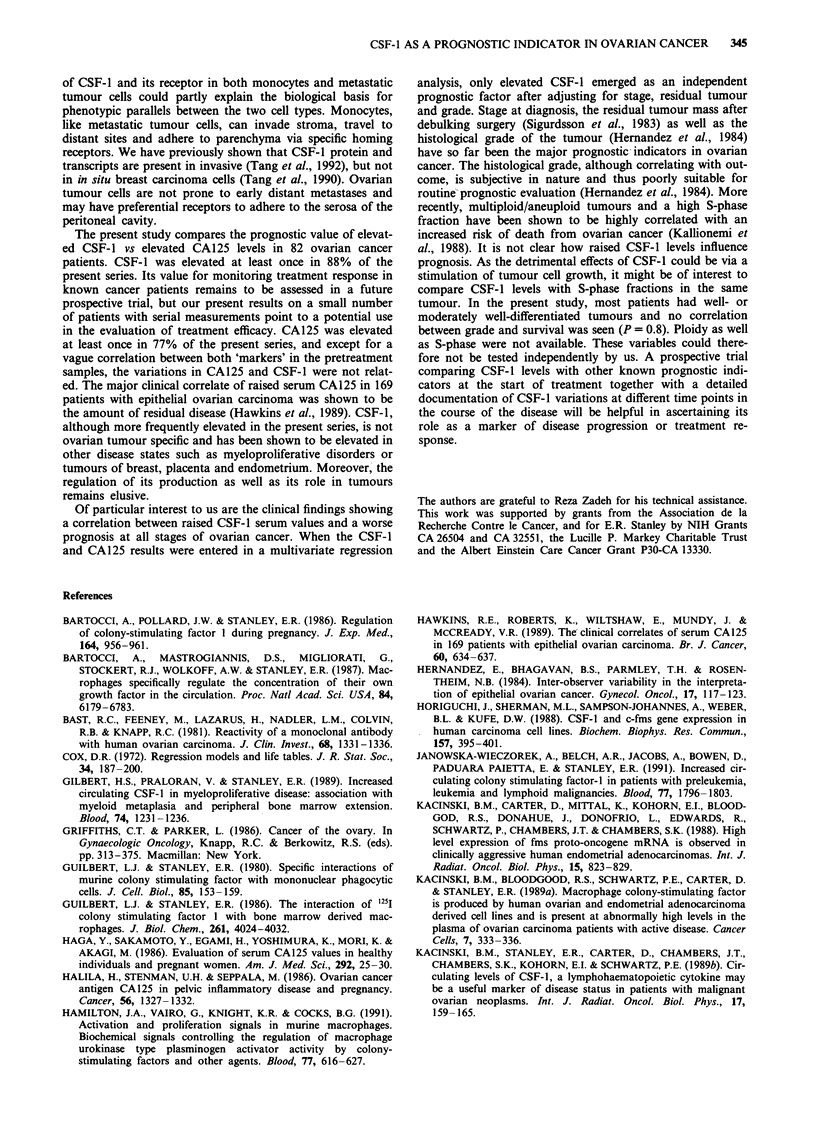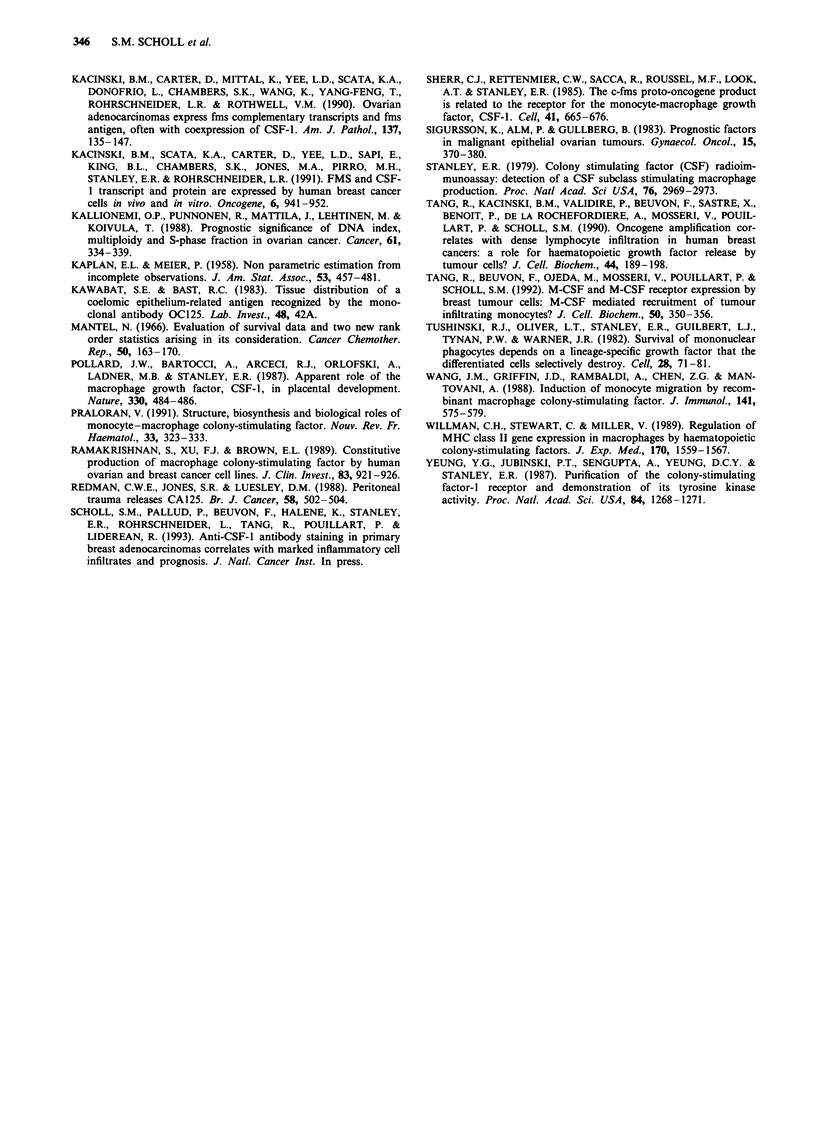# Circulating levels of colony-stimulating factor 1 as a prognostic indicator in 82 patients with epithelial ovarian cancer.

**DOI:** 10.1038/bjc.1994.62

**Published:** 1994-02

**Authors:** S. M. Scholl, C. H. Bascou, V. Mosseri, R. Olivares, H. Magdelenat, T. Dorval, T. Palangié, P. Validire, P. Pouillart, E. R. Stanley

**Affiliations:** Département de Médecine Oncologique, Institut Curie, France.

## Abstract

Serum samples from 82 patients with epithelial ovarian cancer, previously assayed for CA125, were assayed for circulating colony-stimulating factor 1 (CSF-1). An elevated CSF-1 concentration (> 450 U ml-1 or > 5.42 ng ml-1) was significantly associated with a worse survival (P = 0.02). The predictive value of raised CSF-1 levels was retained whether the first available sample for all patients (n = 82) or the first sample at the start of chemotherapy (n = 41) was considered. Mean CSF-1 levels (n = 14) dropped significantly during six courses of platinum-based chemotherapy (P = 0.02). Although an elevated CA125 concentration appeared to be a prognostic indicator in the total population (n = 82), it was not related to prognosis in the group of patients from whom samples had been drawn at the start of chemotherapy. In a Cox proportional hazards model, CSF-1, but not CA125, was significantly associated with outcome following adjustment for stage, grade and degree of surgical clearance.


*Br. J. Cancer* (1994), **69**, 342–346 © Macmillan Press Ltd., 1994

### Circulating levels of colony-stimulating factor 1 as a prognostic indicator in 82 patients with epithelial ovarian cancer

S.M. Scholl[1], C.H. Bascou[1], V. Mosseri[2], R. Olivares[2], H. Magdelenat[3], T. Dorval[1], T. Palangié[1], P. Validire[2], P. Pouillart[1] & E.R. Stanley[4]

[1]*Département de Médecine Oncologique*, [2]*Département de Biostatistiques and* [3]*Département de Pathologie, Institut Curie, 26 rue d'Ulm, 75231 Cedex 05, France;* [4]*Department of Developmental and Molecular Biology, Albert Einstein College of Medicine, 1300 Morris Park Avenue, Bronx, New York 10461, USA.*

**Summary** Serum samples from 82 patients with epithelial ovarian cancer, previously assayed for CA125, were assayed for circulating colony-stimulating factor 1 (CSF-1). An elevated CSF-1 concentration (>450 U $ml^{-1}$ or >5.42 ng $ml^{-1}$) was significantly associated with a worse survival ($P = 0.02$). The predictive value of raised CSF-1 levels was retained whether the first available sample for all patients ($n = 82$) or the first sample at the start of chemotherapy ($n = 41$) was considered. Mean CSF-1 levels ($n = 14$) dropped significantly during six courses of platinum-based chemotherapy ($P = 0.02$). Although an elevated CA125 concentration appeared to be a prognostic indicator in the total population ($n = 82$), it was not related to prognosis in the group of patients from whom samples had been drawn at the start of chemotherapy. In a Cox proportional hazards model, CSF-1, but not CA125, was significantly associated with outcome following adjustment for stage, grade and degree of surgical clearance.

Although progress has been made in establishing meticulous operative staging of ovarian cancer and defining basic principles of therapy, there has not been a dramatic change in survival rates since the 1960s (Griffiths *et al.*, 1986). This bleak outlook is primarily due to the indolent early course of the disease, resulting in an advanced stage at presentation in 70% of all patients. However, even in patients presenting with early disease, an extensive surgical treatment does not guarantee cure and new variables correlating with the malignant potential of the cancer cells would be clinically valuable in the selection of adjuvant therapy for individual patients in early-stage ovarian cancer. Here we report our findings on the predictive value of two tumour 'markers', CA125 and colony-stimulating factor 1 (CSF-1), during the course of ovarian cancer.

CA125 is the antigen recognised by the monoclonal antibody, OC125, produced by immunising Balb/c mice with a cell line, OVCA 433, cultured from the ascitic fluid of a patient with a papillary serous cystadenocarcinoma of the ovary (Bast *et al.*, 1981). Initially it was thought to be specific for ovarian (Kawabat & Bast, 1983) and gastrointestinal malignancies (Haga *et al.*, 1986), but subsequently it was found to be raised in a variety of benign conditions including pregnancy, pelvic inflammatory disease (Haga *et al.*, 1986; Halila *et al.*, 1986) and cirrhosis, suggesting it to be a marker of non-specific peritoneal damage (Redman *et al.*, 1988). CA125 serum levels have since been shown to have a high sensitivity and specificity for predicting response to chemotherapy in ovarian cancer patients as well as for detecting relapse. Most importantly, elevated CA125 levels were found to be correlated with the *amount of residual disease* in a multiple regression analysis (Hawkins *et al.*, 1989).

CSF-1 was originally distinguished from other colony-stimulating factors by its ability to promote survival, proliferation and differentiation of macrophages from bone marrow progenitor cells (Stanley, 1979; Tushinski *et al.*, 1982). Subsequently it was shown to act by binding to and activating a high-affinity membrane tyrosine kinase receptor, the protein product of the oncogene *c-fms* (Guilbert & Stanley, 1980; 1986; Sherr *et al.*, 1985; Yeung *et al.*, 1987). A wide range of non-oncoplastic and tumour cells have since been documented to synthesise CSF-1. These include endometrium, placental trophoblast, endothelial cells, fibroblasts, some T cells, interdigitating reticulum cells and tumours of various origins as well as tumour-derived cell lines (reviewed by Praloran, 1991). Many tumours and tumour-derived cell lines express significant levels of the CSF-1 receptor protein as well, and a possible autocrine role of this growth factor in tumorigenesis has been suggested (Kacinski *et al.*, 1991). The normal steady-state circulating CSF-1 concentration is regulated by sinusoidally located macrophages, which remove the growth factor from the circulation by CSF-1 receptor-mediated endocytosis and intracellularly destroy it (Bartocci *et al.*, 1987). *In vivo* animal studies, using [$^{125}$I]CSF-1 as tracer, showed the half-life of CSF-1 in the circulation to be approximately 10 min (Bartocci *et al.*, 1987). During pregnancy the very high uterine synthesis of CSF-1 may contribute to the slightly elevated circulating CSF-1 concentration (Bartocci *et al.*, 1986; Pollard *et al.*, 1987). Modest elevations in the circulating CSF-1 concentration are evident in disease states such as myeloproliferative disorders (Gilbert *et al.*, 1989; Janowska-Wieczorek *et al.*, 1991) and in ovarian cancer patients, in whom they were highly correlated with the presence of active disease (Kacinski *et al.*, 1989*a*). We set out here to evaluate circulating CA125 and CSF-1 levels during the course of ovarian cancer. We specifically searched for (1) a correlation between these two markers at different time points, (2) the predictive value of elevated levels on patient survival and (3) a change in CSF-1 levels during chemotherapy treatment.

### Materials and methods

### *Patients*

Eighty-two patients (mean age 52) were treated at Institut Curie between 1982 and 1988. Patients' tumours were characterised as follows: all 82 tumours were of epithelial origin (70 serous, two mucinous, five endometrioid, two clear cell and three undifferentiated adenocarcinoma). Staging at diagnosis was: IA, 8; IB, 5; IC, 4; IIA, 5; IIB, 3; IIC, 2; III, 46; IV, 9. One stage IA patient was treated prophylactically because of a strong family history of ovarian, colon and breast cancer; five stage I (A–C) patients had post-surgical chemotherapy for ruptured cyst during the procedure; six stage IA, three stage IB, two stage IC and one stage IIA patients had no chemotherapy. Chemotherapy was administered in 70 patients (85%) and was started 4–5 weeks after primary surgery. Twelve patients were treated for recurrent tumour. Grading was available in 72 patients: 40 (56%) were well differentiated, 28 (39%) moderately well and four (5%) were

Correspondence: S.M. Scholl.
Received 8 December 1992; and in revised form 19 July 1993.

poorly differentiated. Forty-five patients (55%) had a complete surgical resection of their disease; 37 (45%) were incomplete. A second-look operation was carried out in 50 patients (60%) who had a complete clinical and radiological remission following six courses of chemotherapy. Twenty-nine out of 50 (58%) were surgically free of disease, of whom 24 (83%) also had a pathological complete remission. The median follow-up for this patient population was 41 months. Twenty-two out of 82 (27%) patients were alive without recurrence at the time of analysis, in nine disease had recurred and 51/82 (62%) suffered recurrence and died. The median time to recurrence was 27 months for the total population; median survival was 56 months.

The effect of surgery on CSF-1 serum levels was tested in ten patients who had surgery for either malignant (six) or benign (four) disease. Serum samples were collected prior to surgery, immediately after surgery, on day 1 following surgery as well as prior to discharge or at a follow-up visit.

*Serum samples*

Frozen serum samples which had been previously drawn for routine CA125 assay from 82 ovarian cancer patients of Institut Curie were stored at $-30°C$ until use. Radioimmunoassay (RIA) was carried out with iodinated recombinant CSF-1 (a gift from Chiron, Emeryville, CA, USA) and an anti-CSF-1 antiserum as previously described (Janowska-Wieczorek *et al.*, 1991). A total of 420 serum samples were assessed and results are expressed in ng $ml^{-1}$. In a recent study (Janowska-Wieczorek *et al.*, 1991) 64 healthy American controls had normally distributed CSF-1 serum concentrations with a mean concentration of $372 \pm 111$ U $ml^{-1}$, equivalent to $4.48 \pm 1.3$ ng $ml^{-1}$. Serum CA125 concentrations were assayed by solid-phase immunoassay (CA125-EIA, Abbott Park, IL, USA).

For 41 of these patients, samples had been collected at or immediately after primary surgery. Samples were collected from the remaining 41 patients during the periods of initial treatment or observation following treatment, prior to recurrence. Survival curves were drawn according to the first available serum values of CSF-1 and CA125 either for the total group or for the 41 patients for whom samples were collected around the time of primary surgery. Sequential samples at 1, 3 and 6 months of chemotherapy were available on 14 patients, and changes of CSF-1 during the course of chemotherapy treatment were documented for these patients. Fifty patients had a second-look operation. Correlations with CSF-1 and CA125 levels were sought at three different time points during the course of the disease: at the start of chemotherapy, at second look and at recurrence. The predictive value of serum concentrations of CA125 and of CSF-1 at the time of a second-look operation was tested. The relative importance of an elevated CSF-1 concentration *vs* an elevated CA125 concentration as a prognostic indicator was tested in a proportional hazards model as described by Cox (1972).

*Statistical methods*

BMDP programs were run on a VAX 6000 computer. Survival curves were drawn using Kaplan–Meier (Kaplan & Meier, 1958) estimates and comparison of survival distributions was made by log-rank test (Mantel 1966). Serum CA125 and CSF-1 levels were available on the 82 patients at various time points during the course of the disease and survival estimates according to normal/raised levels were calculated according to the first available sample ($n = 82$) for any patient, as well as according to the first sample at the start of chemotherapy ($n = 41$). The cut-off level for CA125 measurements (35 U $ml^{-1}$) had been previously defined as the most discriminant marker of tumour presence (CA125 levels were below 35 U $ml^{-1}$ in 95% normal controls). In the present series 77% of patients had at least one measurement with a value above 35 U $ml^{-1}$.

In an earlier study of American patients with ovarian cancer, a cut-off of 500 U $ml^{-1}$ (6.02 ng $ml^{-1}$) for CSF-1 measurements had been chosen in an attempt to discriminate between the presence or absence of disease and to maximally reduce false-positive (3%) and false-negative (9%) results (Kacinski *et al.*, 1989*b*). Previous studies with normal individuals had indicated that serum CSF-1 concentrations were normally distributed in the range 1.7–7.1 ng $ml^{-1}$ (Gilbert *et al.*, 1989). In the present study, the median value for the 41 patients for whom a serum measurement at the start of chemotherapy was available was 5.42 ng $ml^{-1}$ (450 U $ml^{-1}$), and this value was the most discriminant as a predictor for patient survival (88% of all patients had at least one measurement of CSF-1 above 5.42 ng $ml^{-1}$ during the course of their disease).

Comparison between percentages was by chi-square test, comparison of means by Student's *t*-test. A non-parametric test for paired series (Wilcoxon) was used to compare the values of CSF-1 at the beginning of chemotherapy and 6 months later. The correlation coefficient between CA125 and CSF-1 was calculated at different time points during the course of the disease. The prognostic relevance of CSF-1 and CA125 was adjusted to stage, grade and surgical clearance in a proportional hazards model as described by Cox (1972).

### Results

Serum levels (ng $ml^{-1}$) were measured by RIA and the median value for the group of patients on whom measurements at the start of the first treatment were available ($n = 41$) was 5.42 ng $ml^{-1}$ (450 U $ml^{-1}$). This cut-off value proved discriminant as a predictor for patient survival according to Kaplan–Meier estimates. The CSF-1 concentration in six normal (non-pregnant) French control sera ranged between 0 and 2.6 ng $ml^{-1}$. In 88% of all patients studied, at least one serum measurement was above the cut-off concentration of 5.42 ng $ml^{-1}$, if all samples taken at any time point during the course of the disease are considered. Only 77% of these patients had at least one CA125 measurement that fell above the 35 U $ml^{-1}$ cut-off.

A correlation between elevated CSF-1 and elevated CA125 levels at either the start of chemotherapy (41 patients), at second look (31 patients) or at recurrence (28 patients) was actively sought, but only a vague correlation appeared to exist between these two 'markers' at the start of chemotherapy ($r = 0.3$).

The variation in CSF-1 levels during the course of chemotherapy could be evaluated on 14 patients who had serial samples at 0, 3 and 6 months. A significant decrease, as assessed by a (non-parametric) paired Wilcoxon analysis, was observed after 6 months of treatment ($P = 0.02$ Table Ia). Of these 14 patients, 11 had a complete surgical response; 4/11 were free of microscopic disease. Three patients had no second-look operation, 2/3 had incomplete disease regression and one patient progressed. The variations in CA125 levels during the course of chemotherapy in these patients were

**Table I** (a) Variation in CSF-1 levels during the course of chemotherapy (ng $ml^{-1}$)

| | *Pretreatment level* | *At 3 months* | *At 6 months* |
|---|---|---|---|
| Mean CSF-1 | 7.89 | 5.02 | 2.04 |
| Standard error | 2.07 | 1.06 | 0.44 |

Paired Wilcoxon; variation 0–6 months: $P = 0.02$

(b) Variation in CA125 levels during the course of chemotherapy (U $ml^{-1}$)

| | *Pretreatment level* | *At 3 months* | *At 6 months* |
|---|---|---|---|
| Mean CA125 | 607 | 40 | 27 |
| Standard error | 180 | 28 | 24 |

Paired Wilcoxon; variation 0–6 months: $P = 0.0002$.

highly significant ($P = 0.0002$, Table Ib). The effect of surgery on CSF-1 levels was tested in ten independent patients with benign (four) or malignant (six) disease. Samples were collected prior to and at the end of the procedure, the following day and at discharge or a follow-up visit. No significant changes in CSF-1 serum levels were seen to be associated with surgery (paired Wilcoxon analysis).

Survival curves for those 41 patients for whom a pre-chemotherapy sample was available show a significantly better survival for patients who had 'normal' CSF-1 values (Figure 1), compared with those whose levels were elevated (>5.42 ng $ml^{-1}$; $P = 0.02$). The median survival times were 71 months (<5.42) and 23 months (>5.42) respectively. Tests of the predictive value of elevated CA125 levels on the same samples (before chemotherapy) did not show any difference ($P = 0.38$) and the median survival was 52 and 44 months respectively (Figure 2). However, when data from the first samples of the total population (including patients with recurrent disease) were examined, patients with elevated CA125 levels had a significantly poorer survival ($P = 0.007$); median survival times were 48 and 23 months respectively for patients with normal and with elevated CA125 levels, and those with elevated CSF-1 levels retained the same predictability. The predictive value for combined markers (in 41 patients) is shown in Figure 3. The median survival for those patients who were positive for both 'markers' was 19 months. If either one was positive, the median survival was 52 months; if both were negative, the median survival time was not reached by 60 months ($P = 0.027$).

A total of 50 patients had a second-look operation. Results for both 'markers' at this time point were available on 31 patients; however 34 patients could be assessed according to their CA125 levels alone. Table II shows that elevated CSF-1 levels significantly predicted outcome in those patients who had active disease at second look. The median survival for patients with elevated CSF-1 levels was 17 months; while the median survival (>56 months) was not reached for the group with normal CSF-1 levels. Elevated CA125 values were also significantly predictive of outcome. However, the data were biased by the presence of only two patients with raised CA125 levels who both died early (17 and 20 months).

A multivariate regression analysis comparing the predictive value of raised CSF-1 and CA125 at the start of chemotherapy ($n = 41$) on survival documents a significantly increased risk for patients with elevated CSF-1 values but no increased risk for patients with elevated CA125 levels (Table III).

**Figure 1** Survival according to CSF-1 at start of chemotherapy.

**Figure 2** Survival according to CA125 at start of chemotherapy.

**Figure 3** Survival according to CSF-1 and CA125.

### Discussion

The production of CSF-1 has now been reported in a wide range of tumours of non-hematopoietic origin (Horiguchi *et al.*, 1988; Ramakrishnan *et al.*, 1989; Tang *et al.*, 1990, 1992; Kacinski *et al.*, 1990, 1991; S.M. Scholl *et al.*, submitted). The CSF-1 receptor has also been shown to be expressed in the same tumour types and tumour-derived cell lines, favouring the involvement of CSF-1 in an autocrine mechanism supporting tumour growth. Previous work by Kacinski *et al.* (1990) documents not only the presence of CSF-1 protein and transcripts in ovarian cancer, but also the presence of elevated plasma CSF-1 levels in patients with active and recurrent neoplastic disease (Kacinski *et al.*, 1989*a*).

A number of essential biological functions of monocytes/macrophages, including migration (Wang *et al.*, 1988), production of proteolytic enzymes (Hamilton *et al.*, 1991) and down-regulation of their MHC class II antigen expression (Willman *et al.*, 1989) are inducible by CSF-1. The expression

**Table II** (a) Predictive value of elevated CSF-1 levels (>5.42 ng $ml^{-1}$) at second look

| *Patient group* | n | *>5.42* | *<5.42* | $P^a$ |
|---|---|---|---|---|
| All | 31 | 8 | 23 | 0.07 |
| Pathological remission | 17 | 5 | 12 | 0.46 |
| Active disease | 14 | 3 | 11 | 0.04 |

(b) Predictive value of elevated CA125 levels (>35 U $ml^{-1}$) at second look

| *Patient group* | n | *>35* | *<35* | $P^a$ |
|---|---|---|---|---|
| All | 34 | 2 | 32 | 0.0006 |
| Pathological remission | 19 | | 19 | |
| Active disease | 15 | 2 | 13 | 0.009 |

[a]Log-rank test comparing survival.

(c) Marker value at second look

| | *Sensitivity* | *Specificity* | *PPV*[b] | *At second look* |
|---|---|---|---|---|
| CSF-1 | 3/14 (21%) | 12/17 (71%) | 3/8 (38%) | |
| CA125 | 2/15 (13%) | 19/19 (100%) | 2/2 (100%) | |

[b]PPV, positive predictive value.

**Table III** Cox regression analysis of the risk of death in patients with elevated CSF-1 or CA125 levels at start of chemotherapy

| *Parameter* | *Relative risk* | *Confidence interval (90%)* |
|---|---|---|
| Elevated CSF-1 | 3.23 | 1.2–8.86 |
| Elevated CA125 | 1.95 | 0.69–5.55 |

of CSF-1 and its receptor in both monocytes and metastatic tumour cells could partly explain the biological basis for phenotypic parallels between the two cell types. Monocytes, like metastatic tumour cells, can invade stroma, travel to distant sites and adhere to parenchyma via specific homing receptors. We have previously shown that CSF-1 protein and transcripts are present in invasive (Tang *et al.*, 1992), but not in *in situ* breast carcinoma cells (Tang *et al.*, 1990). Ovarian tumour cells are not prone to early distant metastases and may have preferential receptors to adhere to the serosa of the peritoneal cavity.

The present study compares the prognostic value of elevated CSF-1 *vs* elevated CA125 levels in 82 ovarian cancer patients. CSF-1 was elevated at least once in 88% of the present series. Its value for monitoring treatment response in known cancer patients remains to be assessed in a future prospective trial, but our present results on a small number of patients with serial measurements point to a potential use in the evaluation of treatment efficacy. CA125 was elevated at least once in 77% of the present series, and except for a vague correlation between both 'markers' in the pretreatment samples, the variations in CA125 and CSF-1 were not related. The major clinical correlate of raised serum CA125 in 169 patients with epithelial ovarian carcinoma was shown to be the amount of residual disease (Hawkins *et al.*, 1989). CSF-1, although more frequently elevated in the present series, is not ovarian tumour specific and has been shown to be elevated in other disease states such as myeloproliferative disorders or tumours of breast, placenta and endometrium. Moreover, the regulation of its production as well as its role in tumours remains elusive.

Of particular interest to us are the clinical findings showing a correlation between raised CSF-1 serum values and a worse prognosis at all stages of ovarian cancer. When the CSF-1 and CA125 results were entered in a multivariate regression analysis, only elevated CSF-1 emerged as an independent prognostic factor after adjusting for stage, residual tumour and grade. Stage at diagnosis, the residual tumour mass after debulking surgery (Sigurdsson *et al.*, 1983) as well as the histological grade of the tumour (Hernandez *et al.*, 1984) have so far been the major prognostic indicators in ovarian cancer. The histological grade, although correlating with outcome, is subjective in nature and thus poorly suitable for routine prognostic evaluation (Hernandez *et al.*, 1984). More recently, multiploid/aneuploid tumours and a high S-phase fraction have been shown to be highly correlated with an increased risk of death from ovarian cancer (Kallionemi *et al.*, 1988). It is not clear how raised CSF-1 levels influence prognosis. As the detrimental effects of CSF-1 could be via a stimulation of tumour cell growth, it might be of interest to compare CSF-1 levels with S-phase fractions in the same tumour. In the present study, most patients had well- or moderately well-differentiated tumours and no correlation between grade and survival was seen ($P = 0.8$). Ploidy as well as S-phase were not available. These variables could therefore not be tested independently by us. A prospective trial comparing CSF-1 levels with other known prognostic indicators at the start of treatment together with a detailed documentation of CSF-1 variations at different time points in the course of the disease will be helpful in ascertaining its role as a marker of disease progression or treatment response.

The authors are grateful to Reza Zadeh for his technical assistance. This work was supported by grants from the Association de la Recherche Contre le Cancer, and for E.R. Stanley by NIH Grants CA 26504 and CA 32551, the Lucille P. Markey Charitable Trust and the Albert Einstein Care Cancer Grant P30-CA 13330.

### References

BARTOCCI, A., POLLARD, J.W. & STANLEY, E.R. (1986). Regulation of colony-stimulating factor 1 during pregnancy. *J. Exp. Med.*, **164**, 956-961.

BARTOCCI, A., MASTROGIANNIS, D.S., MIGLIORATI, G., STOCKERT, R.J., WOLKOFF, A.W. & STANLEY, E.R. (1987). Macrophages specifically regulate the concentration of their own growth factor in the circulation. *Proc. Natl Acad. Sci. USA*, **84**, 6179-6783.

BAST, R.C., FEENEY, M., LAZARUS, H., NADLER, L.M., COLVIN, R.B. & KNAPP, R.C. (1981). Reactivity of a monoclonal antibody with human ovarian carcinoma. *J. Clin. Invest.*, **68**, 1331-1336.

COX, D.R. (1972). Regression models and life tables. *J. R. Stat. Soc.*, **34**, 187-200.

GILBERT, H.S., PRALORAN, V. & STANLEY, E.R. (1989). Increased circulating CSF-1 in myeloproliferative disease: association with myeloid metaplasia and peripheral bone marrow extension. *Blood*, **74**, 1231-1236.

GRIFFITHS, C.T. & PARKER, L. (1986). Cancer of the ovary. In *Gynaecologic Oncology*, Knapp, R.C. & Berkowitz, R.S. (eds). pp. 313-375. Macmillan: New York.

GUILBERT, L.J. & STANLEY, E.R. (1980). Specific interactions of murine colony stimulating factor with mononuclear phagocytic cells. *J. Cell. Biol.*, **85**, 153-159.

GUILBERT, L.J. & STANLEY, E.R. (1986). The interaction of $^{125}$I colony stimulating factor 1 with bone marrow derived macrophages. *J. Biol. Chem.*, **261**, 4024-4032.

HAGA, Y., SAKAMOTO, Y., EGAMI, H., YOSHIMURA, K., MORI, K. & AKAGI, M. (1986). Evaluation of serum CA125 values in healthy individuals and pregnant women. *Am. J. Med. Sci.*, **292**, 25-30.

HALILA, H., STENMAN, U.H. & SEPPALA, M. (1986). Ovarian cancer antigen CA125 in pelvic inflammatory disease and pregnancy. *Cancer*, **56**, 1327-1332.

HAMILTON, J.A., VAIRO, G., KNIGHT, K.R. & COCKS, B.G. (1991). Activation and proliferation signals in murine macrophages. Biochemical signals controlling the regulation of macrophage urokinase type plasminogen activator activity by colony-stimulating factors and other agents. *Blood*, **77**, 616-627.

HAWKINS, R.E., ROBERTS, K., WILTSHAW, E., MUNDY, J. & MCCREADY, V.R. (1989). The clinical correlates of serum CA125 in 169 patients with epithelial ovarian carcinoma. *Br. J. Cancer*, **60**, 634-637.

HERNANDEZ, E., BHAGAVAN, B.S., PARMLEY, T.H. & ROSENTHEIM, N.B. (1984). Inter-observer variability in the interpretation of epithelial ovarian cancer. *Gynecol. Oncol.*, **17**, 117-123.

HORIGUCHI, J., SHERMAN, M.L., SAMPSON-JOHANNES, A., WEBER, B.L. & KUFE, D.W. (1988). CSF-1 and c-fms gene expression in human carcinoma cell lines. *Biochem. Biophys. Res. Commun.*, **157**, 395-401.

JANOWSKA-WIECZOREK, A., BELCH, A.R., JACOBS, A., BOWEN, D., PADUARA PAIETTA, E. & STANLEY, E.R. (1991). Increased circulating colony stimulating factor-1 in patients with preleukemia, leukemia and lymphoid malignancies. *Blood*, **77**, 1796-1803.

KACINSKI, B.M., CARTER, D., MITTAL, K., KOHORN, E.I., BLOODGOD, R.S., DONAHUE, J., DONOFRIO, L., EDWARDS, R., SCHWARTZ, P., CHAMBERS, J.T. & CHAMBERS, S.K. (1988). High level expression of fms proto-oncogene mRNA is observed in clinically aggressive human endometrial adenocarcinomas. *Int. J. Radiat. Oncol. Biol. Phys.*, **15**, 823-829.

KACINSKI, B.M., BLOODGOOD, R.S., SCHWARTZ, P.E., CARTER, D. & STANLEY, E.R. (1989*a*). Macrophage colony-stimulating factor is produced by human ovarian and endometrial adenocarcinoma derived cell lines and is present at abnormally high levels in the plasma of ovarian carcinoma patients with active disease. *Cancer Cells*, **7**, 333-336.

KACINSKI, B.M., STANLEY, E.R., CARTER, D., CHAMBERS, J.T., CHAMBERS, S.K., KOHORN, E.I. & SCHWARTZ, P.E. (1989*b*). Circulating levels of CSF-1, a lymphohaematopoietic cytokine may be a useful marker of disease status in patients with malignant ovarian neoplasms. *Int. J. Radiat. Oncol. Biol. Phys.*, **17**, 159-165.

KACINSKI, B.M., CARTER, D., MITTAL, K., YEE, L.D., SCATA, K.A., DONOFRIO, L., CHAMBERS, S.K., WANG, K., YANG-FENG, T., ROHRSCHNEIDER, L.R. & ROTHWELL, V.M. (1990). Ovarian adenocarcinomas express fms complementary transcripts and fms antigen, often with coexpression of CSF-1. *Am. J. Pathol.*, **137**, 135–147.

KACINSKI, B.M., SCATA, K.A., CARTER, D., YEE, L.D., SAPI, E., KING, B.L., CHAMBERS, S.K., JONES, M.A., PIRRO, M.H., STANLEY, E.R. & ROHRSCHNEIDER, L.R. (1991). FMS and CSF-1 transcript and protein are expressed by human breast cancer cells *in vivo* and *in vitro*. *Oncogene*, **6**, 941–952.

KALLIONEMI, O.P., PUNNONEN, R., MATTILA, J., LEHTINEN, M. & KOIVULA, T. (1988). Prognostic significance of DNA index, multiploidy and S-phase fraction in ovarian cancer. *Cancer*, **61**, 334–339.

KAPLAN, E.L. & MEIER, P. (1958). Non parametric estimation from incomplete observations. *J. Am. Stat. Assoc.*, **53**, 457–481.

KAWABAT, S.E. & BAST, R.C. (1983). Tissue distribution of a coelomic epithelium-related antigen recognized by the monoclonal antibody OC125. *Lab. Invest.*, **48**, 42A.

MANTEL, N. (1966). Evaluation of survival data and two new rank order statistics arising in its consideration. *Cancer Chemother. Rep.*, **50**, 163–170.

POLLARD, J.W., BARTOCCI, A., ARCECI, R.J., ORLOFSKI, A., LADNER, M.B. & STANLEY, E.R. (1987). Apparent role of the macrophage growth factor, CSF-1, in placental development. *Nature*, **330**, 484–486.

PRALORAN, V. (1991). Structure, biosynthesis and biological roles of monocyte–macrophage colony-stimulating factor. *Nouv. Rev. Fr. Haematol.*, **33**, 323–333.

RAMAKRISHNAN, S., XU, F.J. & BROWN, E.L. (1989). Constitutive production of macrophage colony-stimulating factor by human ovarian and breast cancer cell lines. *J. Clin. Invest.*, **83**, 921–926.

REDMAN, C.W.E., JONES, S.R. & LUESLEY, D.M. (1988). Peritoneal trauma releases CA125. *Br. J. Cancer*, **58**, 502–504.

SCHOLL, S.M., PALLUD, P., BEUVON, F., HALENE, K., STANLEY, E.R., ROHRSCHNEIDER, L., TANG, R., POUILLART, P. & LIDEREAN, R. (1993). Anti-CSF-1 antibody staining in primary breast adenocarcinomas correlates with marked inflammatory cell infiltrates and prognosis. *J. Natl. Cancer Inst.* In press.

SHERR, C.J., RETTENMIER, C.W., SACCA, R., ROUSSEL, M.F., LOOK, A.T. & STANLEY, E.R. (1985). The c-fms proto-oncogene product is related to the receptor for the monocyte-macrophage growth factor, CSF-1. *Cell*, **41**, 665–676.

SIGURSSON, K., ALM, P. & GULLBERG, B. (1983). Prognostic factors in malignant epithelial ovarian tumours. *Gynaecol. Oncol.*, **15**, 370–380.

STANLEY, E.R. (1979). Colony stimulating factor (CSF) radioimmunoassay: detection of a CSF subclass stimulating macrophage production. *Proc. Natl Acad. Sci USA*, **76**, 2969–2973.

TANG, R., KACINSKI, B.M., VALIDIRE, P., BEUVON, F., SASTRE, X., BENOIT, P., DE LA ROCHEFORDIERE, A., MOSSERI, V., POUILLART, P. & SCHOLL, S.M. (1990). Oncogene amplification correlates with dense lymphocyte infiltration in human breast cancers: a role for haematopoietic growth factor release by tumour cells? *J. Cell. Biochem.*, **44**, 189–198.

TANG, R., BEUVON, F., OJEDA, M., MOSSERI, V., POUILLART, P. & SCHOLL, S.M. (1992). M-CSF and M-CSF receptor expression by breast tumour cells: M-CSF mediated recruitment of tumour infiltrating monocytes? *J. Cell. Biochem.*, **50**, 350–356.

TUSHINSKI, R.J., OLIVER, L.T., STANLEY, E.R., GUILBERT, L.J., TYNAN, P.W. & WARNER, J.R. (1982). Survival of mononuclear phagocytes depends on a lineage-specific growth factor that the differentiated cells selectively destroy. *Cell*, **28**, 71–81.

WANG, J.M., GRIFFIN, J.D., RAMBALDI, A., CHEN, Z.G. & MANTOVANI, A. (1988). Induction of monocyte migration by recombinant macrophage colony-stimulating factor. *J. Immunol.*, **141**, 575–579.

WILLMAN, C.H., STEWART, C. & MILLER, V. (1989). Regulation of MHC class II gene expression in macrophages by haematopoietic colony-stimulating factors. *J. Exp. Med.*, **170**, 1559–1567.

YEUNG, Y.G., JUBINSKI, P.T., SENGUPTA, A., YEUNG, D.C.Y. & STANLEY, E.R. (1987). Purification of the colony-stimulating factor-1 receptor and demonstration of its tyrosine kinase activity. *Proc. Natl. Acad. Sci. USA*, **84**, 1268–1271.